# Macro and Micro-Nutrients Intake, Food Groups Consumption and Dietary Habits among Female Students in Isfahan University of Medical Sciences

**Published:** 2012-04-01

**Authors:** L Azadbakht, A Esmaillzadeh

**Affiliations:** 1 Food Security Research Center, Isfahan University of Medical Sciences, Isfahan, Iran; 2 Department of Nutrition, School of Public Health, Isfahan University of Medical Sciences, Isfahan, Iran

**Keywords:** Dietary intake, Dietary habit, Macronutrient, Micronutrient, Student, Female

## Abstract

**Background:**

Improving the dietary intake among different groups and population is important for improving the health status. This study determines the nutrients and food group intake as well as dietary habits among female students in Isfahan University of Medical Sciences.

**Methods:**

Two hundreds and eighty nine healthy female youths who were randomly selected among students of Isfahan University of Medical Sciences in Isfahan, Iran were enrolled. A validated semi quantitative food frequency questionnaire was used.

**Results:**

Folate, iron, calcium and fiber intake were lower than the recommended dietary allowances (RDA) amounts (70, 76, 90, 56% of RDA, respectively). Forty five percent of the population consumed fast foods 2 times a week and 35% used the frying oils for cooking most of the time.

**Conclusion:**

Female youths had lower amount of some micronutrients. Consuming frying oils, hydrogenated vegetable oils, and fast food intake should be limited among this group.

## Introduction

Considering the dietary intake of different age group of humans is an important issue for assessing the health status,[1] which could help to detect probable nutrient deficiency.[2] Dietary reference intake (DRI) is used to evaluate the diets whether providing enough nutrients to meet requirements.[3] Dietary insufficiencies and malnutrition are associated with some chronic diseases such as osteoporosis, obesity, central adiposity and even type 2 diabetes.[4] Previous studies reported dietary intake of other age groups,[5][6][7] worldwide and in Iran.[8][9]

Micronutrients insufficiency and concerns regarding the diet quality is reported from different countries.[10][11] When looking at the whole diet, it is understood that diet quality of most Tehranian adults[12] and adolescents,[13] needs improvement. Although, some studies discussed the dietary intake, diet quality and dietary patterns in Isfahanian population,[14][15] we were not aware of a recently published paper regarding a comprehensive study including nutrients, and food groups intake besides food habits among Iranian university students. According to the importance of the nutritional status of females in the reproductive age, we determined the dietary intake and habits among female students in Isfahan University of Medical Sciences.

## Materials and Methods

Cluster random sampling method was used to choose a representative sample of female students studding in Isfahan University of Medical Sciences in Iran for the present cross-sectional study. They were in the age range of 18-28 years. Finally, 289 students were enrolled. Written informed consent was obtained from each participant. The study was approved by the Research Council and the Ethical Committee of the School of Health, Isfahan University of Medical sciences.

For assessing the dietary intake, a 168-item semi quantitative food frequency questionnaire was used. We used nutritionist III software (Version 7.0; N-Squared Computing, Salem, OR). Validity and reliability of the FFQ was reported previously which showed good results in this regard.[16] Food habits were assessed by a separated food habit questionnaire. In this food habit questionnaire, dietary habits such as salt intake, kind of oil intake and spices, sugars and sugar sweetened beverages, tea or coffee consumption and frequency of fast food intake were asked from the students.

Anthropometric measurements were done according to the standard method. Systolic and diastolic blood pressure was measured 3 times by a digital instrument and the mean of them was recorded. Descriptive statistics option was used for reporting the mean and standard deviation. Frequency test was used for reporting the prevalence (SPSS Inc., Version 9.05, Chicago, IL, USA).

## Results

Characteristics of the females and the amount of different food group consumption in the present study were shown in Table 1. The mean of body mass index (BMI) was 25.9±5.1 kg/m2. The amount of whole grain and dairy products was lower than recommended amounts based on the guidelines. When we compared the amount of food groups consumed among those students who were living in the dormitory (n=121) and those who were living in their families (n=168), we found no significant difference except for vegetable group which was significantly consumed higher among those living with their families (p=0.04). There were no significant differences regarding any macro and micro nutrients among these two group except for vitamin C intake which was higher among those living with their families (p=0.03). Calorie intake was not different among these two groups.

**Table 1:** Characteristics of the Isfahanian female students and the amount of food groups intake in the present study.

**Variable **	**Min**	**Max**	**Mean±SD**
Age (year)	18	28	21±7
Body mass index (kg/m^2^)	15	31	25.9±5.1
Waist circumference (cm)	60	92	85.5±14.0
Systolic blood pressure (mmHg)	98	133	104±9
Diastolic blood pressure (mmHg)	59	92	72±9
Physical activity (MET. h/wk)	10.1	13.9	12.8±9.7
Food groups			
Partially hydrogenated vegetable oils (gr/day)	10	32	17±2
Non-hydrogenated vegetable oils (gr/day)	9	45	15±1
Fruits (serving /day)	0.5	5	2.2±0.4
Vegetable (serving/day)	0.5	5	3.2±1.0
Meat, Poultary and Fish (serving/day)	0.5	4	2.5±0.5
Whole grains (serving/day)	0	3	1.0±0.2
Refined grains (serving/day)	4	13	6.0 ± 1.2
Legumes (gr/day)	10	40	15 ±3
Low fat dairy (serving/day)	0	2	0.5± 0.11
High-fat dairy (serving/day)	0	2	1.3±0.16

Table 2 shows the macro and micronutrients intake among the Isfahanian female youths. Folate, iron, calcium and fiber intake were lower than the RDA recommended. Dietary habits of the Isfahanian female youths were presented in the Table 3. According to the results, 45% of the population consumed fast foods 2 times a week.

**Table 2:** Daily energy and nutrient intakes of Isfahanian female youths in the study.

**Nutrients**	**Mean±SD**	**RDA^a^**	**% of RDA**
Energy (kcal)	2196±26	-	-
Protein (g)	90±41	46	197
Fat (g)	68±30	-	-
Cholesterol (mg)	154±10	-	-
Saturated fatty acids (g)	24±10	-	-
Polyunsaturated fatty acids (g)	20±8	13	140
Monounsaturated fatty acids (g)	5±0.5	-	-
Carbohydrate (g)	323±100	130	251
Vitamin C (mg)	159±106	75	213
Thiamin (mg)	1.5±0.6	1.1	136
Riboflavin (mg)	2.3±0.8	1.1	211
Niacin (mg)	29±9	14	146
Pyridoxine (mg)	1.6±0.7	1.3	128
Cobalamin (μg)	4.8±0.7	2.4	201
Folate (μg)	310±147	400	77
Vitamin A (μg)	970±63	700	138
Iron (mg)	13±5	18	76
Calcium (mg)	900±503	1000	90
Phosphorus (mg)	1591±597	700	227
fiber (g)	14±1	25	56
Zinc (mg)	9±3	8	113
Selenium (μg)	69±32	55	126

^a^ RDA: Recommended Dietary Allowances.

**Table 3:** Dietary habits of Isfahanian female youths in the study.

**Dietary habits**	**Percent**
Tea or coffee	
Tea (750 ml or less)	50
Tea (more than 750 ml)	50
Coffee (120 ml or more)	2
Fast foods	
Fast food consumption (never or one time a week)	50
Fast food consumption (two times a week)	42
Fast food consumption (more than 2 times per week)	8
Salty snacks (more than 2 times per week)	16
High fat snacks (more than 2 times per week)	49
Different kind of oil	
Non-hydrogenated vegetable oils (every day)	51
Hydrogenated vegetable oils (every day)	75
Specific oils for frying (every day)	35
Animal oils (every day)	3
Beverages	
Sugar sweetened drinks (every day)	36
Colas	30
Sugars	
Sugars with drinking tea (more than 10 g a day)	74
Spices (commonly used during a week)	
Vinegar	3
Lemon juice	37
mayonnaise and high fat salad sauces	56
Mostly method of cooking during a week	
Boiling and steaming	28
Frying	68
Grilling	4
Break fast consumption	
Every day	25
Not regularly (2 times a week or lower)	23
Never	52

## Discussion

The results of the present study which was conducted among Isfahanian female youths revealed that the amount of folate, iron, and calcium and fiber intake was lower than the recommended amounts by RDA. This population had lower amount of dairy intake compared to the guidelines. Furthermore, some dietary habits such as consuming frying oils, hydrogenated vegetable oils, sugar sweetened beverages and fast food intake should be improved among them. The mentioned items were the key findings of this study.

There were some previous reports regarding the dietary intake of youths worldwide.[17] Low intake of calcium, iron, zinc, vitamin A was reported from secondary school in England.[17] The age of the population in the present study was older than them. However, the results are similar to some extent. The most problematic micronutrients among women in Burkina Faso were vitamin B-12, folate (12%), riboflavin (13%), and niacin (20%).[18] Although the present study was also conducted on females but the age range of the present study was younger than them. Results from the present study on female are similar to the results from Burkina Faso. Results from the related studies in Iran showed low intake of calcium, iron and vitamin B series11 in adults. Low dairy intake has been emphasized in previous studies in Iran among adults.[19] In the present study, dairy intake was lower than the recommended amounts. Besides the nutritional benefits of dairy consumption, sufficient dairy intake is an important issue for preventing non-communicable diseases and improving the health status especially in the developing countries. Furthermore, lower dairy intake is associated with lower quality of the diets.[19] Fruit and vegetable intake was also in the lowest amount of recommended range. The amount of refined grain consumption was extremely higher than the whole grain in this population. Most popular Iranian traditional dishes are full of white rice.10,11 Fiber intake from the source of whole grain could have beneficial effects for health. So, it seems that nutrition education programs are needed in this area especially for the young adults in Iran. Previous studies revealed that nutrition education could improve the dietary intake and could provide a healthy eating pattern.[20] Using an appropriate educational model could play an important role for achieving the best results.[20]

Among dietary habits, fast food consumption was high among this population. Fast foods are a major source of trans fat intake. The results of a recent analysis of different fast foods in Iran revealed a high percentage of trans fats content of them.[21] Analysis of the dietary intake has shown that Iranians consume twice as much TFAs as the US population.[22] High amount of saturated fatty acids in fast foods are also a major concern. Other studies also revealed that fast food intake is a major problem in the diet of young people.[23][24] According to the results, high percentage of Isfahanian female youths consumed frying oils and hydrogenated oils which were major sources of trans fats. Focusing on the omega-3 fatty acids consumption, it is an important issue in the field of dietary intake among young people.[25] Kelishadi et al. also showed that a high percent of Iranian children and adolescents (73.8% of families) consumed hydrogenated fats based on the results of the CASPIAN study.[26] The results derived from the present study are in line with the results from Kelishadi et al.

Fifty two percent of the female youths did not consume breakfast in the present study. Skipping breakfast is reported in other countries as well and the results from the present study which is similar to most previously published papers.[26][27] Breakfast eating pattern is similar among children, adolescents and even young adults. Nutrition education programs focusing on breakfast eating pattern could be beneficial in this regard.

In the present study, we had both students from the university dormitory and also students who were living with their families. So, we had this opportunity to have a representative sample of female students in Isfahan. This was more confirmed by using a stratified randomized sampling method. However, the results are specified to the female university students and not to all females in Iran. This might decrease the external validity of the study. However, we had different socioeconomic categorize of the university students in our study. We used a validated semi-quantitative food frequency questionnaire which would be related to lower rate of bias. However, some degree of measurement error in the assessment of dietary intake by a FFQ is inevitable as is in all dietary assessment methods.

In conclusion, the key findings of the current report revealed that the amount of folate, iron, calcium and fiber intake among Iranian female youths was lower than the recommended amounts by RDA. This population had lower amount of dairy intake compared to the guidelines. Furthermore, some dietary habits such as consuming frying oils, hydrogenated vegetable oils, and fast food intake should be improved among them.
